# Effect of pretreatment on physicochemical, microbiological, and aflatoxin quality of solar sliced dried ginger (*Zingiber officinale* Roscoe) rhizome

**DOI:** 10.1002/fsn3.1878

**Published:** 2020-09-13

**Authors:** Roseline Esi Amoah, Faustina Dufie Wireko‐Manu, Ibok Oduro, Firibu Kwesi Saalia, William Otoo Ellis

**Affiliations:** ^1^ Ghana Standards Authority Accra Ghana; ^2^ Department of Food Science and Technology Kwame Nkrumah University of Science and Technology Kumasi Ghana; ^3^ Department of Food Process Engineering University of Ghana Legon Ghana

**Keywords:** drying, ginger, microbial, potassium metabisulfite, pretreatment, rhizome

## Abstract

Pretreatment of fruit and vegetables is necessary to reduce microbial proliferation and to preserve color of the produce. The effect of drying and pretreatment with potassium metabisulfite (KMBS) of concentrations 0.0%, 0.1%, 0.15%, 0.2%, and 1.0% and blanching at 100°C and 50°C using a tent‐like concrete solar (CSD) dryer as compared to open‐sun drying (OSD) of yellow ginger rhizomes was investigated using routine methods. The total color change and residual sulfur dioxide (SO_2_) were analyzed. KMBS reduced the yeast and mould load significantly from 3.6 × 10^4^ ± 1.4 × 10^3^ CFU/g in 0.0% (control) to <10 CFU/g in 1.0% KMBS and 100°C blanched fresh samples. Drying of the fresh samples for 5 days increased the yeast and mould load of all the treatments to as high as 1.15 × 10^5^ ± 2.12 × 10^4^ CFU/g for the 1.0% KMBS. Overall, the CSD had fewer microbial loads than the OSD but it was not significant. Aflatoxins and *Salmonella* sp. were not detected in any of the samples. The sulfur dioxide residue (SO_2_) for KMBS pretreated samples increased as the concentration of KMBS increased with the CSD retaining slightly higher amount than the OSD. The total color change index increased with increase in KMBS, and drying further increased the total color change index. On the whole, the blanched samples had the least color change among the pretreatments with 100°C CSD showing the least change among the dried samples.

## Graphical abstract

Pretreatment of ginger rhizome with increasing concentration of potassium metabisulfite reduced microbial load significantly while drying increased it again. Blanching of the ginger slices at 1,000 C had minimum color deviation and concrete solar dried ginger preserved the color better.

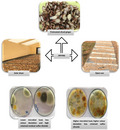

## 1 INTRODUCTION

Pretreatments are widely used before drying of agro‐products to inactivate enzymes, enhance drying process, and improve quality of dried products (Lizhen et al., 2017). These may involve the application of both chemicals and physical treatments. Some chemical treatments used which may either be liquid or gaseous may include hyperosmotic solutions such as sucrose, alkali liquors (sodium hydroxide (NaOH), sodium chloride (NaCL), potassium hydroxide (KOH), sulfites (sodium metabisulfites (NaMBS), and potassium metabisulfites (KMBS)), and others such as ethyl oleate (EO) and sulfur dioxide (SO_2_). Physical approaches may include various forms of thermal blanching (hot water, steam, super‐heated steam etc) and nonthermal treatments (ultrasound and freezing) (Lizhen et al., 2017). Drying is the process of reducing the moisture content of an agricultural produce to an acceptable limit by the application of heat to reduce microbial proliferation and increase shelf‐life of the produce (Sansaniwal & Kumar, 2015). Several drying methods are used for the drying of different produce (An et al., 2015; Ding, An, Zhao, Guo, & Wang, 2012). However, in tropical regions, open‐sun drying is the most widely used (Fudholi, Ruslan, Othman, Zaharim, & Sopian, 2013) due to its economic viability (Sansaniwal & Kumar, 2015). Open‐sun drying however has many disadvantages such as high microbial load, vast color changes to produce, and contamination by foreign matter such as sand and pieces of twigs and leaves from neighboring flora. Solar drying on the other hand could be a good alternative (Deshmukh, Varma, Yoo, & Wasewar, 2014; Fudholi et al., 2013). Studies have shown that different solar dryers with different designs affect the drying time and color of a particular produce. Ginger rhizome (*Zingiber officinale* Roscoe) is an important cash crop of the world that can be used in the fresh and dried form, both as spice and for its medicinal properties on a daily basis. It has been reportedly used, as a pain relief for arthritis, muscle soreness, chest pain, lower back pain, stomach pain, and menstrual pain (Gümü Say, Borazan, Ercal, & Demirkol, 2015; Shukla & Singh, 2007). The importance of this spice underscores it drying for year‐round availability. Prasad, Prasad, and Vijay (2006) in drying ginger using solar biomass hybrid dryer and open‐sun drying reported that the solar dried samples took less drying time of 33 hr while the open‐sun dried took 96 hr to dry. Deshmukh et al. (2014) using a mixed mode box‐cabinet natural circulation solar dryer with an average temperature of 57 ± 8.5°C in the drying of ginger reduced its initial moisture content of 621.50% to 12.19% (d.b.) within 8 hr while open‐sun drying took 2–3 days. The evaluation of total change in color showed that the solar dried ginger rhizome had less color change than the open‐sun dried. To improve quality of produce, pretreatment of produce prior to drying or processing is considered as an alternative. This process helps to reduce microbial load, improves organoleptic properties, and has a synergetic effect in improving the shelf‐life of the produce. Pretreatments using potassium metabisulfite (KMBS) and blanching have been reported to reduce microbial load and also preserve the color of ginger. Sulfite is used as an additive in the food industries for its numerous benefits such as inhibiting enzymatic and nonenzymatic browning, increasing antioxidant properties of the food product to prevent oxidative spoilage; (oxygen scavenger and reducing agent) and inhibiting the action of enzymes such as proteases, oxidases, peroxidases, as well as an antimicrobial and a fungistat. Sulfite also plasmolyzes cells which facilitate drying (Latapi & Barrett, 2006a, 2006b; Onyemaobi & Williams, 2012; Sangwan, Kawatra, & Sehgal, 2012).

*Campylobacter* and *Escherichia coli* O157:H7 outbreaks have been reported as well as *Salmonella* sp. contamination of fresh herbs (Li et al., 2017). Research has also shown that fresh ginger contains high loads of moulds such as *Aspergillus flavus* (Ramesh & Santoshkumar, 2013; Singh, Gitansh, & Bhadauria, 2013; Toma & Abdulla, 2013) which has the potential to produce aflatoxins in the dried ginger product. This thus requires proper treatment and monitoring of aflatoxin development at different doses (Jeswal & Kumar, 2015; Rajarajan, Rajasekaran, & Devi, 2013). Even though studies have established the benefits of pretreatments and solar drying, it is produce specific in terms of the type and conditions of the solar dryer and pretreatment. The objective of this study was to assess the effect of pretreatment and drying using a tent‐like concrete solar dryer on the physicochemical and microbiological quality of sliced solar dried ginger rhizome compared to open‐sun drying.

## 2 MATERIALS AND METHODS

### 2.1 Source of raw materials

Eighty kilograms (80 kg) of fresh ginger rhizomes of 9 months maturity were purchased from an out‐grower in the ginger‐producing areas of the Ashanti region of Ghana. The fresh ginger rhizomes were transported to the laboratory packed in perforated nylon sack (aeration).

### 2.2 Washing and pretreating the fresh ginger

Fresh raw ginger was soaked in water for 2 hr to remove the adhering sand/debris and washed vigorously three times each time with fresh water. The washed ginger rhizomes were sliced manually with a kitchen knife to a thickness of 3–5 mm. The sliced ginger was washed again in water. The sliced ginger was divided into seven parts; four parts were soaked separately in 0.1%, 0.15%, 0.2%, and 1.0% potassium metabisulfite (KMBS) concentration for 5 min. Another one part was soaked in water for 5 min serving as the control (0.0% KMBS concentration). The final two parts were soaked in water at 50°C and 100°C temperature for 5 min and 60 s, respectively. All the pretreated sliced ginger rhizomes were drained separately and dried.

### 2.3 Drying of the fresh washed sliced ginger rhizome

The different pretreatments were drained separately and sampled for physicochemical, microbial and aflatoxin analysis as well as sulfur dioxide (SO_2_) and color determination. The remaining sliced fresh ginger of KMBS and blanching pretreatment were divided into two. One part was dried with the concrete solar dryer (CSD) and the other one part dried using the open sun (OSD) for 5 days and subjected to same analysis as in the fresh. Temperature and humidity of the environmental conditions were recorded using a digital temperature‐humidity data logger (HOBO pro v2 digital logger (Model U23‐001)). Temperature and humidity ranged from 29.0–52.5°C; 27%–91.5% RH and 28.0–55.5°C; 29.5%–90.5% RH for the CSD and OSD, respectively (Appendix 1, Appendix 2, and Appendix 3).

#### 2.3.1 Milling of ginger samples

The samples (fresh washed or dried ginger) were pulverized into paste and powder, respectively, using a Philips mill (HR 2113/05).

### 2.4 Physicochemical analysis

#### 2.4.1 Moisture content

The moisture content determination followed the method of AOAC (1980). Moisture cans were heated in the hot air oven for 1 hr, cooled in a desiccator and weighed. Five grams of the pulverized/milled fresh washed and dried ginger was weighed into the conditioned moisture dish and spread evenly. The moisture dish containing the sample was placed in the oven and heated for three (3) hours at 105°C. The dish containing the sample was placed in the desiccator to cool and then reweighed. The percentage moisture content was calculated as below:

$$\frac{\left(\text{wt}\text{. of can}+\text{sample}\right)‐\left(\text{wt}\text{. of empty can}\right)\times 100}{\text{Wt}\text{. of sample taken}}=\%$$

#### 2.4.2 Total ash/acid‐insoluble ash content

The analysis was done according to AOAC (1980). Two grams of the milled fresh washed or dried ginger sample was weighed into a previously preheated, cooled, and weighed crucible. The sample was then decarbonized on a Bunsen burner. The crucible containing the sample was placed in the furnace at 600°C for 3 hr. After 3 hr, the crucible was cooled in a desiccator and weighed again and difference in weight was calculated as total ash content. To the total ash obtained as described above, 20 ml of 10% hydrochloric acid (HCl) was added and boiled gently for 5–10 min on a hot plate. The sample was quantitatively transferred into a filter paper in a funnel on a beaker. The residue from the crucible was washed with boiling distilled water into the filter paper. This was washed severally with hot distilled water until the filtrate was free of chloride ions. The filter paper was dried in the oven at 105°C ± 2°C for 30 min. The filter paper containing the acid‐insoluble ash was decarbonized on a Bunsen burner and placed in a furnace at 550°C for 1 hr. The crucible was cooled in a desiccator and weighed to the nearest 0.1 mg.

Calculation (Total ash)

$$\frac{\left(\text{wt}.\;\text{of crucible}+\text{ash}\right)‐\left(\text{wt}.\;\text{of empty crucible}\right)\times 100}{\text{Wt}.\;\text{of sample taken}}=\%$$

Acid‐insoluble ash

$$\frac{\left(\text{wt}.\;\text{of crucible}+\text{acid}\;‐\;\text{insoluble ash}\right)‐\left(\text{wt}.\;\text{of empty crucible}\right)\times 100}{\text{Wt}.\;\text{of sample taken}}=\%$$

wt. ‐ weight.

### 2.5 Sulfur dioxide residue analysis

The sulfur dioxide residue (dry weight basis) was determined according to the modified method by Reith Williams (FAO, 1986; Owureku‐Asare, Oduro, Saalia, Tortoe, & Ambrose, 2018). Twenty‐five grams of fresh ginger or dried milled ginger was dispersed in 20 ml of water and diluted with 25 ml of dilute sodium hydroxide. It was allowed to stand for 5 min and diluted with 10 ml sulfuric acid. The mixture was allowed to stand for another 5 min, and 1 ml of starch indicator added. It was titrated with standard iodine solution to a permanent purple color.

### 2.6 Color measurement

The tristimulus color of sliced fresh, solar, and open‐sun dried ginger rhizome was measured using the Minolta Chroma meter (CR‐410, Konica Minolta Optics Inc). The surface color in terms of lightness (*L*″‐value), redness (*a*″‐value), and yellowness (*b*″‐value) of sliced fresh, solar, and open‐sun dried ginger rhizome was measured. The total color change (Δ*E*) was calculated from the differences in *L*″, *a*″, and *b*″ values using the equation below;

$$\Delta E=\surd \Delta L^{2}+\Delta a^{2}+\Delta b^{2}$$

### 2.7 Microbiological analysis

#### 2.7.1 Quantitative estimation of fungal population

The fungal population of the fresh and dried ginger samples was determined using the spread plate method described in ISO 21527: (2008)‐1&2. Thirty grams of the ginger sample was transferred into 500‐ml conical flasks containing 270 ml of 0.1% peptone water as diluent. Each flask was shaken at 140 rpm for 20 min on an Orbital shaker. Serial dilution up to 1:10^9^ was made, and 0.1 ml aliquots were inoculated in sterile Petri dishes containing already poured Dichloran Rose Bengal Chloramphenicol agar (DRBC) for fresh ginger and Dichloran‐18‐Glycerol agar (DG 18) for dried samples. All the DRBC and DG18 plates were incubated at 28 ± 2°C for 5–7 days. Plates containing fungal colonies were counted, and the population expressed as CFU/g sample.

#### 2.7.2 Determination of *Salmonella* sp. contamination

This followed the procedure described in ISO 6579: (2002). Twenty‐five grams of ground ginger (fresh or dried) was added to 225 ml of peptone water and shaken for 20 min. The culture was incubated at 37°C ± 1°C for 24 ± 3 hr. An aliquot of 0.1 ml of the culture was taken and added to 10 ml of Rappaport‐Vassiliadis Soya Peptone (RSV) broth. The mixture was incubated at 41.5 ± 1°C for 24 ± 3 hr. This culture was then plated on bismuth sulfite agar at 37°C ± 1°C for 24 ± 3 hr.

Colonies with black centre and a lightly transparent zone of reddish color or pink with a darker centre were subcultured in nutrient broth and incubated at 37°C ± 1°C for 24 ± 3 hr. A little of the incubated culture was then stabbed and streaked on TSI agar and incubated at 37°C ± 1°C for 24 ± 3 hr. A red slant and yellow butt indicated the presence of *Salmonella* sp. Results were expressed as presence or absence of *Salmonella* sp.

### 2.8 Aflatoxin analysis

#### 2.8.1 Extraction and clean‐up

Extraction and clean‐up are based on ISO method 16050‐2003. A mixture of ground sample (20 g) with 2.0 g of sodium chloride and 100 ml of methanol/deionized water (80:20) was homogenized (Ultra Turrax homogenizer (Ultra Turrax type and/or blender with a capacity of 1 L, equipped with aluminum cover, operating at high speed) at high speed for 3 min and filtered through Whatman No. 541 filter paper. The extract (20 ml) was diluted with 60 ml of phosphate buffer and filtered through a 1.0 µm glass microfiber filter. The diluted extract (20 ml) was passed through Vicam Aflatest Immunoaffinity column (IAC), which was washed twice with 10 ml deionized water. The aflatoxin was eluted from the IAC with 2 ml high‐pressure liquid chromatography (HPLC)‐grade methanol into amber glass vials, and 50 µl of the eluent was injected into the HPLC.

#### 2.8.2 HPLC determination

HPLC determination was done based on ISO method 16050‐2003 with a Kobra Cell for post‐column derivatization. A C‐18 column (200 × 4.6 mm, 5 µm) with Shimadzu 20A series coupled with florescence detector was used. The mobile phase used was methanol:water (40:60, v/v) at a flow rate of 1.2 ml/min with column temperature maintained at 40°C ± 1°C. To 1 L of mobile phase were added 119 mg of potassium bromide and 350 μl of 4 M nitric acid (required for post‐column electrochemical derivatization with Kobra Cell, R‐Biopharm Rhone). Calibration curve of reference aflatoxin Mix Standards (G_1_, G_2_, B_1_, B_2_) from Romer Labs ® was prepared with the maximum concentration of 12.5 ng/ml in methanol. Limit of detection and limit of quantification for total aflatoxin were established at 0.1 µg/kg, and a recovery rate of G1, G2, B1, and B2 was 64%, 81%, 65%, and 82%, respectively.

### 2.9 Statistical analysis

The data for all analysis conducted in triplicates were subjected to analysis of variance (ANOVA), SAS^®^ JMP Pro 13 test at *p* ≤ .05 to determine significant differences between treatments for the samples. Multiple range analysis was done using Tukey's HSB.

## 3 RESULTS AND DISCUSSION

### 3.1 Effect of potassium metabisulfite and blanching on physicochemical quality of ginger

The results of the physicochemical analysis are shown in Table 1. Increase in the concentration of % KMBS was relatively proportional to residual sulfur dioxide before and after drying. In other words, as the concentration of KMBS increased, the residual sulfur dioxide also increased. Fresh ginger samples recorded the highest residual sulfur dioxide (77.08 ± 1.50–724.58 ± 25.40 ppm) followed by solar dried (32.45 ± 0.06–47.13 ± 0.17 ppm) and then open‐sun dried samples (35.19 ± 0.02–46.83 ± 0.08 ppm). The difference in the residual sulfur dioxide of the fresh samples compared to the residual SO_2_ of both dried samples was significant; however, between the dried samples the difference was not significant even though the solar dried samples retained more. Similar findings were reported by Okzan and Cemeroglu (2002), and Latapi and Barrett (2006a, 2006b), of the concentration of residual SO_2_ during drying or heat treatment. They reported the reduction of the initial residual SO_2_ from 2,444 ppm to 1,587 ppm.

**TABLE 1:** Physicochemical analysis of fresh and solar dried pretreated ginger on dry weight basis

Name of sample	Treatments	*Moisture content (%)	Total ash (%)	Acid‐insoluble ash (%)	Sulfur dioxide residue (ppm)	Change in color (Δ*Ε*)
Fresh samples	KMBS conc					
	0.0%	82.20 ± 0.97^a^	7.83 ± 0.07^a^	0.80 ± 0.07^ab^	77.08 ± 1.5^a^	–
	0.10%	83.19 ± 0.27^a^	7.63 ± 0.08^a^	1.03 ± 0.16^a^	81.78 ± 0.04^a^	1.07 ± 0.23^a^
	0.15%	82.91 ± 0.78^a^	7.45 ± 0.61^a^	0.84 ± 0.08^ab^	100.92 ± 0.25^a^	2.99 ± 0.13^b^
	0.2%	82.50 ± 0.22^a^	7.87 ± 0.23^a^	0.75 ± 0.03^ab^	98.45 ± 0.37^a^	5.56 ± 1.08^d^
	1.0%	81.43 ± 0.36^a^	6.71 ± 0.28^a^	0.64 ± 006^ab^	724.58 ± 25.4^b^	7.77 ± 2.29^e^
	Blanching					
	50.0°C	82.22 ± 0.04^a^	6.45 ± 0.01^a^	0.52 ± 0.02^b^	N/A	1.68 ± 0.85^a^
	100.0°C	83.72 ± 0.26^a^	7.07 ± 0.97^a^	0.77 ± 0.12^ab^	N/A	1.03 ± 0.39^a^
Solar dried	KMBS conc					
	0.0%	10.03 ± 0.23^b^	7.19 ± 0.18^a^	0.80 ± 0.05^ab^	32.45 ± 0.06^c^	–
	0.10%	9.16 ± 0.07^c^	7.27 ± 0.21^a^	0.85 ± 0.12^ab^	36.96 ± 2.9^c^	6.13 ± 0.79^de^
	0.15%	8.52 ± 0.02^c^	7.19 ± 0.07^a^	0.73 ± 0.07^ab^	38.78 ± 0.09^c^	5.02 ± 0.10^cd^
	0.2%	10.52 ± 0.17^b^	7.86 ± 0.51^a^	0.76 ± 0.02^ab^	39.82 ± 0.01^c^	5.18 ± 0.35^cd^
	1.0%	10.45 ± 0.05^b^	7.59 ± 0.08^a^	0.89 ± 0.0^ab^	47.13 ± 0.17^c^	5.53 ± 0.15^d^
	Blanching					
	50.0°C	9.28 ± 0.16^c^	7.32 ± 0.18^a^	0.64 ± 0.02^b^	N/A	4.23 ± 0.50^bcd^
	100.0°C	8.21 ± 0.06^cd^	7.36 ± 0.06^a^	0.94 ± 0.11^ab^	N/A	3.44 ± 0.30^b^
Open‐sun dried	KMBS conc					
	0.0%	10.01 ± 0.03^b^	7.19 ± 0.05^a^	0.77 ± 0.01^ab^	35.19 ± 0.02^c^	–
	0.10%	8.92 ± 0.1^cd^	7.38 ± 0.09^a^	0.73 ± 0.03^ab^	35.54 ± 0.02^c^	5.60 ± 0.19^cd^
	0.15%	9.18 ± 0.34^c^	7.71 ± 0.13^a^	0.93 ± 0.16^ab^	35.81 ± 0.0^c^	5.10 ± 0.13^cd^
	0.2%	9.49 ± 0.02^c^	7.45 ± 0.2^a^	0.62 ± 0.01^ab^	39.51 ± 0.05^c^	7.38 ± 0.61^e^
	1.0%	9.12 ± 0.11^c^	7.49 ± 0.09^a^	0.75 ± 0.02^ab^	46.83 ± 0.08^c^	11.25 ± 0.77^f^
	Blanching					
	50.0°C	9.09 ± 0.07^c^	7.57 ± 0.19^a^	0.91 ± 0.19^ab^	N/A	4.73 ± 0.99^bc^
	100.0°C	8.57 ± 0.1^cd^	7.31 ± 0.04^a^	0.75 ± 0.01^ab^	N/A	5.87 ± 0.36^d^

Note

Different alphabets as superscript in the same column denote significance.

N/A—not applicable; the blanched samples were not treated with KMBS.

* Moisture content of the fresh samples was calculated on wet basis.

In a study conducted by Okzan and Cemeroglu (2002), on the effect of the removal of SO_2_ from apricot by hot air drying at different temperatures, the results showed that as the temperature and time increased, more SO_2_ was removed. The initial SO_2_ content of dried apricots of 4,577 ± 28:4,836 ± 63:4,005 ± 54 mg/kg dry wt after 96 hr of drying at 40°C, 50°C, and 60°C, respectively, dropped by 19.6%, 27.6%, and 64.2%, respectively.

Generally, the appropriate moisture content of a product is important in preventing microbial proliferation (Deshmukh et al., 2014) and change in flavor and color. The drying techniques used in this study effectively reduced initial moisture content from 80% to 10% (d.b) over the drying period of 5 days (Table 1). Temperature and humidity ranged from 29.0–52.5°C; 27%–91.5% RH and 28.0–55.5°C; 29.5%–90.5% RH for the CSD and OSD, respectively (Appendix 1, Appendix 2, and Appendix 3). The solar dried samples recorded a moisture content of 8.21 ± 0.06 to 10.03 ± 0.23% while the open‐sun dried samples had values from 8.57 ± 0.1 to 10.01 ± 0.03% d.b (Table 1). The KMBS pretreatment and blanching at 50°C for 5 min and 100°C for 60 s did not affect the moisture content of the fresh ginger samples significantly neither were the dried samples affected. The total ash content of ginger determines the age and maturity of the product. The total ash content of the 9‐month‐old ginger used in this study recorded less than 8.0% for all the samples. Also, pretreatment did not significantly affect the total ash (%) of the ginger for fresh samples (6.45 ± 0.01–7.87 ± 0.23 d.b), solar dried (7.19 ± 0.18–7.86 ± 0.51 d.b), and open‐sun dried (7.19 ± 0.05–7.71 ± 0.13 d.b.) (Table 1).

The acid‐insoluble ash (AIA) content of a product measures the amount of sandy matter a product contains. Open‐sun dried products are usually implicated of being less hygienic because of the open environment (Fudholi et al., 2013). In this study, the least AIA content of 0.52 ± 0.02% for the fresh sample was also seen as having the least AIA content (0.64 ± 0.02%) in the CSD while KMBS treated 0.2% sample had the least AIA of 0.62 ± 0.01% for the OSD. The fresh, CSD and OSD recorded AIA values as high as 1.03 ± 0.16%, 0.94 ± 0.11%, and 0.93 ± 0.16 respectively, which are comparable. It may be due to the fact that the environment used for the open‐sun drying in this study was clean preventing the OSD samples from having higher sandy matter than the CSD and fresh. Thus, the environment used for open sun drying affects the quality of a product.

The cosmetic quality of food is influenced by color which is a primary evaluating attribute accessed by consumers (Calvo, 2004). The color in a food product during handling is influenced by naturally occurring pigments resulting from both enzymatic and nonenzymatic reactions (Marshall, Kim, & Wei, 2000). In this study, the change in color of the treated samples was 1.03 ± 0.39–7.77 ± 2.29 for fresh samples, 3.44 ± 0.30–6.13 ± 0.79 for the solar dried samples, and 5.10 ± 0.13–11.25 ± 0.77 for the open‐sun dried samples. With the exception of open‐sun dried samples, blanching at 100°C prior to drying resulted in minimum change in color. The total color change index increased significantly with increase in KMBS concentration which may be due to the increasing sulfite. This result agrees with the findings of Latapi and Barrett (2006a, 2006b) that increasing the KMBS concentration for tomato pretreatment affected the color significantly. Apart from the 0.1% KMBS CSD samples, the trend was similar for the dried samples (increasing % KMBS showing increasing change in color index) which may be due to the amount of residual SO_2_ concentration. The low change in color in this study occurred in 0.1% KMBS treated fresh samples and both blanched samples of the fresh ginger which may be attributed to the ability of the treatment regimens to delay or prevent the oxidative reaction initiated by the polyphenol oxidase in enzymatic browning as well as the type of drying (Ioannou & Ghoul, 2013). These results also showed that drying itself affected the color; the fresh 100°C with the initial total color change of 1.03 ± 0.39 had increased to 3.44 ± 0.30 for CSD and 5.87 ± 0.36 for OSD. Generally, the CSD samples had minimum total color change as compared to the OSD irrespective of the treatment employed. The 1.0% KMBS treated samples recorded a total color change index of 5.53 ± 0.15 for the CSD and 11.25 ± 0.77 for the OSD which was significant. This finding agrees with the findings of Deshmukh et al. (2014) who showed that total color change index value of solar dried ginger yielded a better quality as compared to open sun drying. The total color change index value in their work was found to be 31.05 and 52.32 for solar and open sun‐dried ginger, respectively.

Blanching inhibits enzyme activity and, subsequently, browning. This was evident in this study as blanched samples for the fresh and both drying methods except the 100°C OSD had less total color change as compared to the KMBS treated samples.

### 3.2 Effect of potassium metabisulfite and blanching on microbial quality of ginger

The microbial quality of the samples is shown in Table 2. The results showed that there was a general decrease in microbial load (yeast and mould) with increasing potassium metabisulfite (KMBS) for both the fresh samples and the dried samples. For all the samples, *Salmonella* species were absent. Potassium metabisulfite (KMBS) is a chemical that reduces microbial load in different concentrations of food produce, and in this study, it is used as the pretreatment agent. For the fresh samples, there was a significant general decrease in microbial load from 3.60 × 10^4^ for the control (0.0% KMBS) to less than 10 for the 1.0% KMBS (Table 2). Even though the general trend was significant, this was not the trend for the extent of decrease between 0.1% and 0.15% KMBS concentration. The same pattern is observed for the solar dried samples with a general decrease in yeasts and moulds from a load of 9.10 × 10^4^ (control‐0.0% KMBS) to 4.75 × 10^4^ CFU/g (1.0% KMBS) but not significant.

**TABLE 2:** Microbiological quality and total aflatoxin content in fresh and solar dried pretreated ginger

Name of sample	Treatments	Yeast & mould (CFU/g)	*Salmonella* sp.	Total aflatoxin content (ppb)
Fresh samples	KMBS concentration			
	0.0%	3.60 × 10^4^ ± 1.41 × 10^3de^	Absent	ND
	0.10%	2.93 × 10^3^ ± 5.77 × 10^1e^	Absent	–
	0.15%	1.50 × 10^2^ ± 7.07 × 10^1e^	Absent	–
	0.2%	<10^fe^	Absent	–
	1.0%	<10^fe^	Absent	–
	Blanching			
	50.0°C	6.53 × 10^3^ ± 1.80 × 10^3e^	Absent	–
	100.0°C	<10^fe^	Absent	–
Solar dried	KMBS concentration			
	0.0%	9.10 × 10^4^ ± 2.83 × 10^3cde^	Absent	ND
	0.10%	6.40 × 10^4^ ± 5.66 × 10^3cde^	Absent	ND
	0.15%	5.30 × 10^4^ ± 4.24 × 10^3cde^	Absent	ND
	0.2%	4.95 × 10^4^ ± 7.07 × 10^2cde^	Absent	ND
	1.0%	4.75 × 10^4^ ± 3.54 × 10^2cde^	Absent	ND
	Blanching			
	50.0°C	1.63 × 10^4^ ± 4.35 × 10^2de^	Absent	ND
	100.0°C	1.7 × 10^5^ ± 2.83 × 10^2e^	Absent	ND
Open‐sun dried	KMBS concentration			
	0.0%	2.05 × 10^6^ ± 1.27 × 10^5a^	Absent	ND
	0.10%	1.29 × 10^5^ ± 6.44 × 10^4cd^	Absent	ND
	0.15%	1.56 × 10^5^ ± 4.08 × 10^4c^	Absent	ND
	0.2%	8.08 × 10^4^ ± 1.57 × 10^4cde^	Absent	ND
	1.0%	1.15 × 10^5^ ± 2.12 × 10^4cde^	Absent	ND
	Blanching			
	50.0°C	1.82 × 10^6^ ± 4.50 × 10^3e^	Absent	ND
	100.0°C	1.38 × 10^4^ ± 2.83 × 10^4b^	Absent	ND

Note

Different alphabets as superscript in the same column denote significance (*p* ≤ .05).

Abbreviations: –, not analyzed; ND, not detected.

The same observation is made with the open‐sun drying (OSD) showing a general decrease in microbial load from 2.95 × 10^6^ CFU/g (control) to 1.15 × 10^5^ CFU/g (1.0% KMBS). However, for OSD, there was a slight variation between 0.2% and 1.0% where 0.2% had a slightly lower microbial load (8.08 × 10^4^ CFU/g) compared to 1.15 × 10^5^ CFU/g for 1.0% KMBS. The general observation would have been to have a lower value for 1.0% and a higher value for 0.2%. However, statistical analysis indicates that the difference was not significant (*p* > .05) (Table 2). In terms of load content, the fresh samples had relatively lower microbial load compared to the dried samples. Thus, the period of drying could have increased the microbial load of the sliced ginger irrespective of the drying and pretreatment methods used. The fresh control (0.0%) with an initial load of 3.60 × 10^4^ increased to 9.10 × 10^4^ CFU/g for the CSD which was not significant (*p* > .05) and to 2.05 × 10^6^ CFU/g for the OSD which was significant (*p* < .05). Thus, the CSD had less load (9.10 × 10^4^ CFU/g) compared to OSD (2.05 × 10^6^ CFU/g). Blanching at 50°C for 5 min did not have a significant reduction in microbial load when compared to the control (0.0%). However, the 100°C blanched fresh ginger for 60 s recorded almost no growth (Table 2) implying that the heat and time were sufficient to kill the microbes. From the results of this study, it can be inferred that the following pretreatment conditions: 0.2% KMBS, 1.0% KMBS, and 100°C blanching for 60 s were ideal for the processing of fresh ginger rhizome. The significant increase of the microbial load in the different KMBS pretreatments for CSD may be due to the reduction in the residual concentration of SO_2_ (Table 2) (Latapi & Barrett, 2006a, 2006b; Onyemaobi & Williams, 2012) and the high relative humidity of both drying methods (Appendix 1, Appendix 2, and Appendix 3). This is because studies have shown that a relative humidity higher than 70% allows growth of most moulds (Fact Sheet, 2019; Ibrahim, Rabah, Liman, & Ibrahim, 2011). This notwithstanding, the CSD samples showed less microbial population than the OSD samples. This agrees with the findings of Eze and Agbo (2011), and that solar dried samples had lower microbial load (2.18 × 10^5^ CFU/g) than open‐sun dried samples (2.6 × 10^5^ CFU/g). However, the yeast and mould load recorded in this study were higher than reported by Eze and Agbo (2011). It is also higher than the mould load recorded by Addo (2005), and Ahene, Odamtten, and Owusu (2011), who showed that ginger, harbored a fungal population ranging from 2.4 to 3.0 log10 CFU/g sample. The presence of *Salmonella enterica* sp. is an indication of poor handling and environmental filth and poses a public health importance because of its virulence nature. Studies have reported the presence of *Salmonella* sp. on dried spices and herbs (Sagoo et al., 2009) which is responsible for salmonellosis for foods that contained spices. The absence of *Salmonella* sp. in the ginger used in this study thus alleviates the potential of such health hazards.

Even though this work did not specifically identify the type of mould species present, reports from other studies by Ramesh and Santoshkumar (2013), Singh et al. (2013), and Jeswal and Kumar (2015) have shown that the most dominant genera of mycoflora with mycotoxigenic potential in their studies of five different spices was *Aspergillus* (7 species) followed by *Penicillium* with 3 species, *Fusarium* with 2 species, and *Mucor* with a species. In their studies, the *Aspergillus* species had the aflatoxin producing *A. flavus* and *A. parasiticus* and *A. niger*, *A. ochraceus* which produces ochratoxin.

Results of this study showed the absence of aflatoxin (Table 2), and this may possibly be attributed to the absence of virulent strains of aflatoxin producing mycoflora on the ginger rhizome; it may also be due to the ability of the drying techniques used to rapidly dry the ginger slices, thus preventing the creation of conducive environment for toxin production. Studies have also reported the antimicrobial activities of ginger (Sa‐Nguanpuag, Kankyanarat, Sriloang, Tanprasert, & Techavuthporm, 2011; Singletary, 2010), and this may have also hindered the production of aflatoxins by the associated moulds. This notwithstanding, a few studies have shown aflatoxin content lower than the allowable limits of 10 ppb in ginger while some findings have also shown levels of aflatoxins higher than 10 ppb in ginger (Menon & Zavier, 2010; Mwangi, Nguta, & Muriuki, 2014). Rajarajan et al. (2013) and Thirumala‐Devi et al. (2001) in their works recorded aflatoxin levels of 23 and 80 mg/kg and 15.5–25 μg/kg, respectively. The high aflatoxin contamination by these researchers may be due to prolonged drying period, high humidity during drying, or improper storage facilities which increases the relative humidity and moisture content to levels for mould growth and aflatoxin production.

## 4 CONCLUSION

The drying methods reduced the moisture of the ginger rhizome from an initial level of 80% wet base to 10% d.b in 5 days. Increasing the concentration of KMBS did not affect the moisture content in this study as expected or the total ash content of ginger. Total color change, however, increased with increasing KMBS concentration, and 0.1% KMBS preserved the color of the fresh samples better because high concentrations of KMBS increase bleaching and consequently a higher total color deviation. However, 100°C blanching was best for all pretreated ginger in preserving color because it inactivated the enzymes that causes browning. Among the dried samples, 100°C blanching and CSD had less total color change. The effect of potassium metabisulfite (KMBS) application for the sliced fresh ginger rhizome reduced significantly the yeast and mould load as the concentration of (KMBS) increased. For the dried samples, there was an increase in the yeast and mould load. However, the solar dried samples (CSD) had fewer loads than the open‐sun dried (OSD) samples. Even though the yeast and mould load were relatively high, aflatoxins were not detected in any of the dried samples for both CSD and OSD which will prevent a possible aspergillosis and mycotoxin infections.

The study shows that increased concentrations of KMBS reduce microbial load, and therefore, the food industry can employ a higher concentration than 1.0% to increase the acidity of the produce and prevent microbial proliferation during drying.

## CONFLICT OF INTEREST

The authors declare that they do not have any conflict of interest.

## ETHICAL APPROVAL

This study does not involve any human or animal testing.
